# VECTOS: An Integrated System for Monitoring Risk Factors Associated With Urban Arbovirus Transmission

**DOI:** 10.9745/GHSP-D-18-00300

**Published:** 2019-03-22

**Authors:** Clara B. Ocampo, Neila J. Mina, Maria I. Echavarria, Miguel Acuña, Alexi Caballero, Andres Navarro, Andres Aguirre, Ingrid S. Criollo, Francia Forero, Oscar Azuero, Neal D. Alexander

**Affiliations:** a Centro Internacional de Entrenamiento e Investigaciones Médicas (CIDEIM), Santiago de Cali, Colombia.; b Red AEDES, Bucaramanga, Colombia.; c Universidad ICESI, Santiago de Cali, Colombia.; d Corporación para la investigación de la Corrosión (CIC), Piedecuesta Santander, Colombia.; e Municipal Secretariat of Health from Yopal, Yopal Colombia.; f Municipal Secretariat of Health form Giron, Giron, Colombia.; g Municipal Secretariat of Health from Buga, Buga, Colombia.

## Abstract

In Colombia, as in many Latin American countries, decision making and development of effective strategies for vector control of urban diseases such as dengue, Zika, and chikungunya is challenging for local health authorities. The heterogeneity of transmission in urban areas requires an efficient risk-based allocation of resources to control measures. With the objective of strengthening the capacity of local surveillance systems to identify variables that favor urban arboviral transmission, a multidisciplinary research team collaborated with the local Secretary of Health officials of 3 municipalities in Colombia (Giron, Yopal, and Buga), in the design of an integrated information system called VECTOS from 2015 to 2018. Information and communication technologies were used to develop 2 mobile applications to capture entomological and social information, as well as a web-based system for the collection, geo-referencing, and integrated information analysis using free geospatial software. This system facilitates the capture and analysis of epidemiological information from the Colombian national surveillance system (SIVIGILA), periodic entomological surveys—mosquito larvae and pupae in premises and peridomestic breeding sites—and surveys of knowledge, attitudes, and practices (KAP) in a spatial and temporal context at the neighborhood level. The data collected in VECTOS are mapped and visualized in graphical reports. The system enables real-time monitoring of weekly epidemiological indicators, entomological indices, and social surveys. Additionally, the system enables risk stratification of neighborhoods, using selected epidemiological, entomological, demographic, and environmental variables. This article describes the VECTOS system and the lessons learned during its development and use. The joint analysis of epidemiological and entomological data within a geographic information system in VECTOS gives better insight to the routinely collected data and identifies the heterogeneity of risk factors between neighborhoods. We expect the system to continue to strengthen vector control programs in evidence-based decision making and in the design and enhanced follow-up of vector control strategies.

## Abstract (precis)

To strengthen local surveillance of mosquito-borne viral diseases such as dengue and Zika, a multidisciplinary team developed an integrated web-based information system called VECTOS that captures geo-referenced entomological, epidemiological, and social data. The system has revealed previously unidentified features, such as specific neighborhoods, at persistently high risk.

## INTRODUCTION

*Aedes (Stegomyia) aegypti* and *Ae. albopictus* mosquitoes transmit viruses including dengue, chikungunya, and Zika, principally in urban environments where there is a concentration of larval habitats, especially artificial water containers, and an abundance of human hosts within the relatively short flight range of the *Aedes* vectors.^1^ Dengue is considered to be the most important arbovirus, with an estimated 390 million infections per year, of which 96 million are symptomatic^2^ and around 24,000 are fatal.^3^ Chikungunya and Zika were introduced to the Americas in 2014 and 2015, respectively, resulting in outbreaks that affected most countries in the region.^4^^,^^5^ Other arboviruses, such as yellow fever, Mayaro, and Oropouche are emergent or re-emergent health threats.^4^^,^^6^

Prevention and control of these diseases focus largely on the vector. However, vector control is often reactive and largely relies on insecticides, which are expensive and decreasingly effective due to resistance.^7^ Additionally, the complexity and granularity of urban areas requires a better understanding of local risk factors to focus vector control actions.^8^^–^^12^ Factors associated with transmission include sociodemographic characteristics, household conditions, proximity among houses, water supply, solid waste, drainage systems, and lack of knowledge of the disease.^12^^–^^14^ The adult mosquitoes tend to bite during the day, their flight is often facilitated by open unscreened doors and windows, and they frequently find ample wet containers to lay their eggs. The use of new tools to capture detailed information on risk factors and to analyze data in real time could strengthen surveillance and monitoring systems by informing local vector control and educational programs, including early outbreak actions.^15^^–^^18^

The development of tools based on geographic information systems (GIS) and information and communication technologies lowers barriers to multivariable analysis and identification of spatial and environmental patterns of transmission risk. These analyses have been shown to contribute to the design of targeted strategies with public health impact.^19^^–^^21^ Some countries—including Brazil,^22^ Argentina,^23^ Mexico,^24^ and Sri Lanka^25^^,^^26^—have developed technological strategies for the evaluation of indicators of transmission risk. Most of these systems are designed mainly to collect information at the municipality level and not at a granular scale, such as the neighborhood, which is important for dengue due to the short flight range of the *Aedes* vectors.^27^

In Colombia and most other Latin American countries (exceptions being Brazil and Mexico), local evidence-based decision making is limited and delayed by paper-based data collection and non-automated data analysis at a coarse level (municipality). In Colombia, no technological tools are officially in use yet, although some initiatives are beginning to be developed to collect the information to carry out real-time analysis over space and time in order to produce integrated analyses of available surveillance information.

Here we describe the lessons learned from the development of the integrated web-based (online) information system called VECTOS, which we developed following Colombia's national surveillance guidelines. The main goal is to empower local health officials to monitor risk factors for the transmission of urban arboviruses, and hence facilitate design and evaluation of focused evidence-based strategies for their prevention and control.

## PROGRAM DESCRIPTION

**FIGURE 1:**
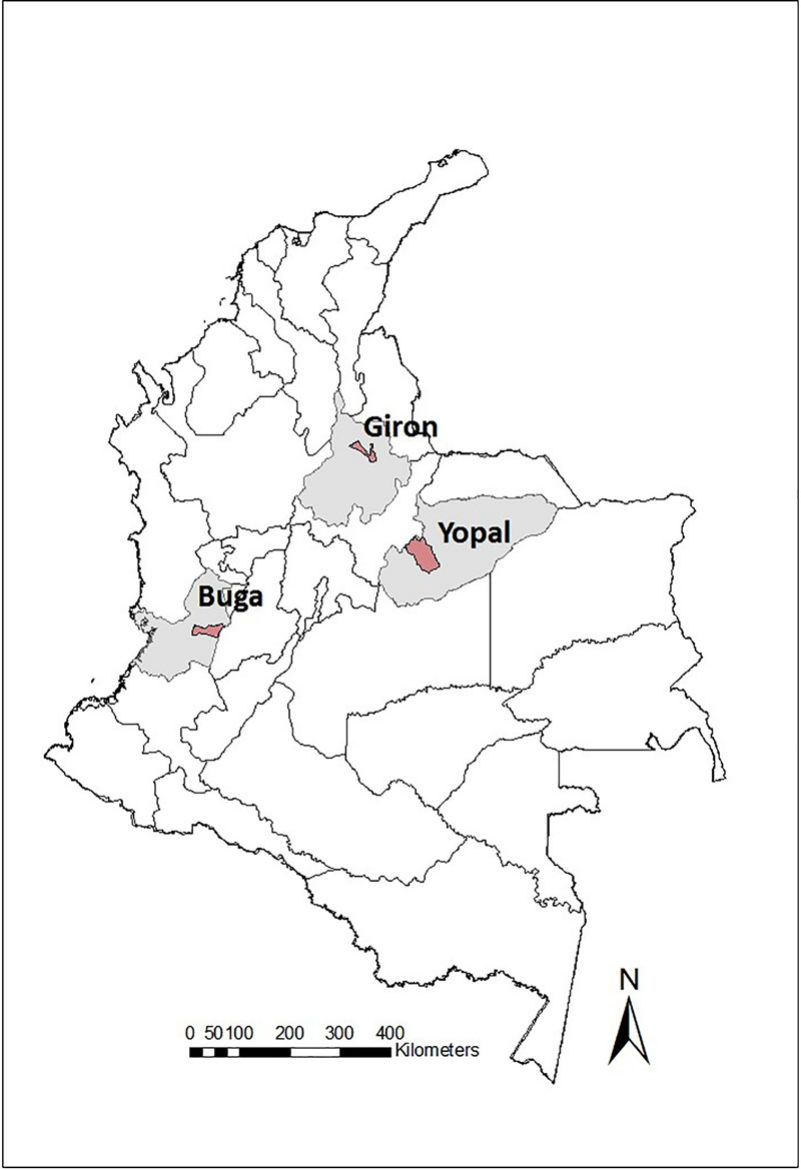
Departments (gray) and Municipalities (red) in Colombia participating in the Study, 2015–2018

The VECTOS information system was developed, from 2015 to 2018, by a team of researchers (epidemiologists, entomologists, anthropologists, and systems engineers) working together with the officials of the Municipal Health Secretariat (health professionals and technicians) of Casanare, Santander, and Valle del Cauca. One municipality in each of 3 departments (states) of Colombia that participated in the project was selected: Yopal in Casanare, Giron in Santander, and Buga in Valle del Cauca (Figure 1). These municipalities are of medium size (100,000 to 150,000 inhabitants) and similar in terms of dengue incidence (336–587 cases per 100,000 habitants per year),^28^ health care, and access systems. Each has an epidemiologist or statistician, and a vector-borne disease program (in Spanish *Enfermedades Transmitidas por Vectores* or ETV), with a coordinator and a relatively stable team of 5 to 10 technicians.

The design of the VECTOS web system was based on the characterization of routine data collection and analysis by the Municipal Health Secretariat and the Colombian national guidance on epidemiology and entomology. These data were analyzed based on city maps and demographic information. Different stakeholders were contacted for information on patient data collection, analysis, and procedures and to share these data with other governmental entities. Based on group analysis and literature, a set of variables from patient and entomological information was used to create the preliminary web system and the reports to facilitate analysis. Improvements to the algorithms for geo-referencing the data, to the analysis report pages, and to the web interface were made throughout the study. The final purpose of the web system was to have a user-friendly spatial tool to gather and analyze data that would meet user needs in real time. This allowed the use of historic epidemiological data already computerized and the creation of mobile applications to gather entomological and social data.

VECTOS was developed to use this routinely collected information, hence avoiding a redundant parallel system and creating additional burden on local staff. Although VECTOS is scalable to other municipalities, the following information describes its products to date.

### The VECTOS Integrated Model Structure

**FIGURE 2:**
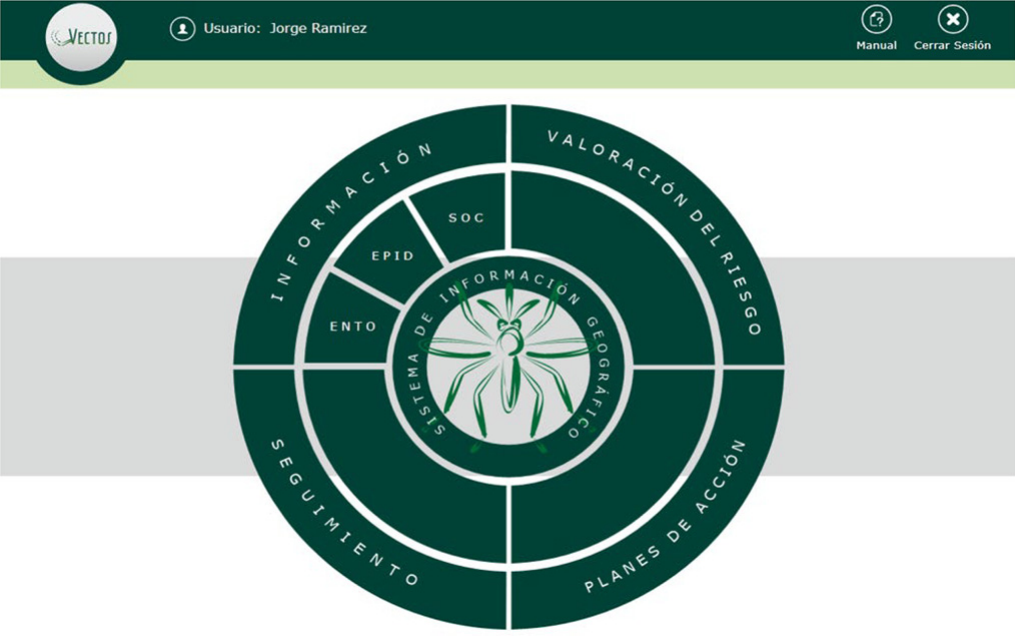
The Initial VECTOS Menu (“Splash Screen”) Options From top left: *Información* (Information) leads to data visualization and analysis of entomological (*Ento*), epidemiological (*Epid*), or sociological (*Soc*) data. *Valoración del riesgo* (Risk assessment) allows the user to create and visualize risk maps. *Planes de acción* (Action plans) allows the user to organize and schedule field activities. *Seguimiento* (Follow-up) allows the user to monitor progress on the action plans. In the center: *Sistema de información geográfica* (Geographic information system) shows the geo-referenced raw data.

VECTOS captures epidemiological, entomological, and social data at the neighborhood level, which can be aggregated by commune (*comuna*) administrative division or sector. It is a web-based information system with a service-oriented architecture that offers robustness in terms of stability and security access and flexibility when interacting with other systems. It was developed in Microsoft Silverlight 5.0 in the C programming language. It has 3 interacting layers. First, the graphical interface or presentation layer is the front-facing system with which the user interacts (Figure 2). The second layer contains the business logic that establishes the rules to be fulfilled. Finally, the data access layer interacts with the database and delivers the data to the business logic layer.

#### Global Architecture

The system is made up of the following 3 components, listed in increasing level of user interaction.

- **Database management system:** This component contains the main database, configurations, and administrative tools, and ensures traceability.
- **Web application:** This is an application program that is stored on a remote server and delivered over the Internet through a browser interface. This fulfills clients' requests via visualization on browsers (e.g., Internet Explorer, Mozilla Firefox), and communicates with the database to obtain data for items such as analysis, models, and reports.
- **Clients:** These consisted of the terminals, computers, or devices with web browsers that support Hypertext Markup Language (HTML) and active content from the web application.

#### Administration Module

Specified users can perform actions in the administration module, such as configuring the system, creating users, and assigning roles and access privileges (for example, ability to run analyses and export data vs. to only visualize reports). These configurations define a security scheme based on the use of roles for navigation within the system.

#### Geographical Display

VECTOS presents geographical data in layers of: (1) political-administrative division; (2) epidemiological, entomological, and social data; and (3) risk stratification. Maps of neighborhoods were created by digitizing physical maps using QGIS 2.18.3, an open-source geographic information software, and validating the maps with a municipal planning secretariat official. These maps, as well as their aggregates at the *Comuna* (sector) level, were plotted using Google Street Map as a base.

#### Field Data Collection

We developed 2 mobile applications (“apps”) to capture entomological and social data in the field, called Mosquitos and VECTOS Social, respectively.

- **Mosquitos app:** This app was developed for Android 4.4.2 Gingerbread for compatibility with low-cost devices and hence increased accessibility. It was developed and tested in collaboration with field technicians to ensure its acceptability.
- **VECTOS Social app:** This captures sociodemographic data including surveys of knowledge, attitudes, and practices (KAP) regarding dengue and its control, using a questionnaire developed by an anthropologist. The app was developed for Android 4.4 Xapp.

The 2 mobile applications capture entomological and social data in the field to transmit in real time to VECTOS server, including automatic geo-location, via hypertext transfer protocol (http). In case of network failure, the data are stored and synchronized when mobile phone or Wi-Fi connectivity is restored. Both apps are available in Google Play Store, but they currently require authorization to connect to data in the web system.

Two mobile applications capture entomological and social data in the field to transmit to VECTOS.

### Data Acquisition and Reports

#### Demography

Neighborhood population size was obtained from the Secretary of Municipal Planning where possible. Otherwise, it was estimated as the number of premises with a water distribution point registered with the water supply company in that neighborhood, multiplied by a factor of 5 people per premise, based on data from the entomological surveys. Other sources, such as community organizations, were also consulted. In the case of recent unplanned developments without registered water supply, we consulted the Colombian Identification System for Potential Beneficiaries of Social Programs.

#### Epidemiology

Epidemiological data are collected weekly by local health institutions (known as the Primary Data Generating Unit) and reported to the Colombia National System of Public Health Surveillance (Sistema Nacional de Vigilancia en Salud Pública – SIVIGILA). From the multiple variables collected by SIVIGILA, and with previous agreement with the local Secretary of Health officials, we selected key variables to be incorporated in VECTOS for epidemiological analysis such as identification of epidemiological patterns of arboviral cases, early detection of outbreaks, stratification of transmission risk at the neighborhood level, as well as for monitoring adherence to protocols and surveillance processes in health care institutions.

SIVIGILA variables captured by VECTOS can be grouped into 3 categories:

1. Basic event (disease) characterization, for example, illness event code, occurrence and reporting date, epidemiological week, name of the Primary Data Generating Unit and municipality reporting the case, and neighborhood, area, sector, municipality, and department of origin of the event
2. Patient demographics, such as age, gender, pregnancy status, residential address, neighborhood, area, sector, and municipality of residence
3. Clinical case features, management, and outcomes, such as date of consultation, date of onset of symptoms, initial and final classification of the case, hospital management of the patient, lab tests and results, and patient's final condition and case report updates

VECTOS captures information from health institutions about disease characterization, patient demographics, and clinical case features, management, and outcomes.

The VECTOS algorithm follows the routine clerical process of the local health secretariats. Weekly reports of 5 notifiable events (dengue, severe dengue, dengue mortality, Zika, and chikungunya) since 2008 have been uploaded to VECTOS. Reported cases represent symptomatic patients who consult at health care centers. Dengue cases are usually classified initially as probable, pending laboratory results. For Zika and chikungunya, cases are usually classified as suspected or confirmed based on clinical diagnosis. The VECTOS system can receive uploads containing the cumulative cases reported to SIVIGILA during the year, in order to reflect possible updates to case status based on lab results. These uploads are currently done manually in the form of Microsoft Excel reports generated by SIVIGILA. This could potentially be automated in the future should national-level approval be obtained to link the systems.

Additionally, to locate each reported case at the neighborhood and sector level for spatial and temporal analysis, another algorithm was developed to geo-reference the cases' residential addresses (although this is not necessarily the locus of transmission). This algorithm was designed to cope with misspellings and abbreviations in the address and neighborhood fields. It was designed to learn from accumulating data and so improve over time. The system attempts to locate each address within the GIS polygon of the corresponding neighborhood, and this was achieved for approximately 84% of the cases.

VECTOS includes an algorithm that cross-references reported cases with their residential address, which was achieved for 84% of cases.

**FIGURE 3:**
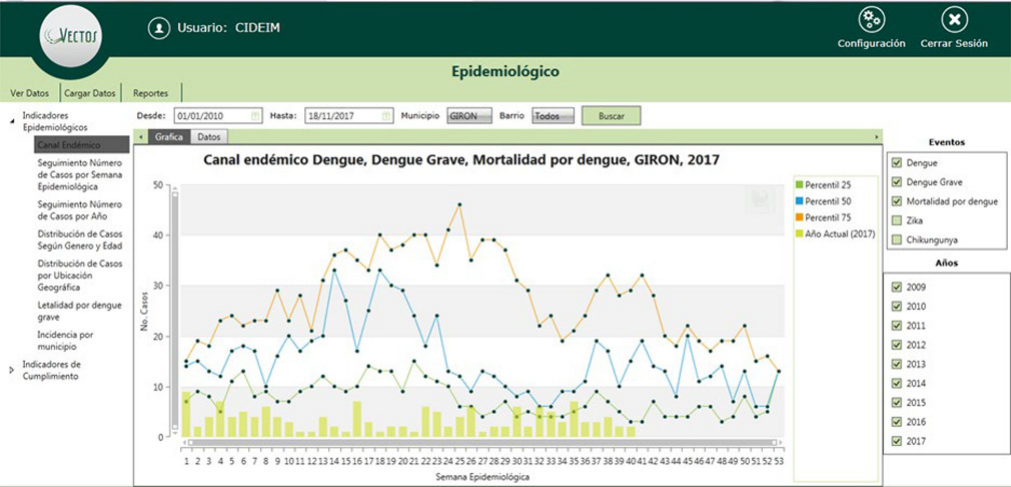
VECTOS Graphical Report of Epidemiological Indicators This example shows actual data from the municipality of Giron in 2017. More specifically, the dengue endemic channel is indicated by the lines, which are percentiles of case numbers, from January 1, 2010, to November 18, 2016. Bars show the contemporaneous data for 2017.

Standard epidemiological indicators are included, such as endemic channel (i.e., the expected normal seasonal range)^29^; number of cases per week and year; distribution of cases by gender, age, and geographic location; fatality rate; and incidence by municipality and neighborhood. Graphical epidemiological reports of spatial and temporal analyses can be carried out automatically for a specified time period, municipality, neighborhood, or event (Figure 3). These reports were designed to effectively convey the local epidemiological situation and facilitate decision making. At the request of the local Secretary of Health, in order to monitor the processes of surveillance and patient care, additional indicators have been included, such as hospitalization rates and the classification of cases as suspected, probable, or confirmed.

#### Entomology

A household survey of potential *Aedes* larval habitats, using the Mosquitos app, was developed based on Colombian surveillance guidelines.^30^ This survey, carried out by the field ETV technicians, collects the following information: (1) type of larval habitat (i.e., type of container, such as high rooftop water storage tanks, low [ground-level] water storage tanks in wash or laundry basins, water storage containers, tires, flower pots and vases, bottles or cans, natural breeding sites, small miscellaneous [<500 ml] and large miscellaneous [>500 ml] containers); (2) presence of immature stages of *Aedes spp*; (3) number of *Aedes* pupae; (4) presence of *Culex* larvae; (5) some household demographic information; and (6) the state of sewage and trash collection services. The app also collects information on potential breeding sites in public areas—such as catch basins (roadside storm drains), stormwater runoff channels, and waste disposal—to identify potential peridomestic larval habitats. In order for the pupae count to be operationally feasible, it was agreed with the technicians to count up to 30 pupae and thereafter give an approximate calculation.

All the *Stegomyia* indices (house index, Breteau index, container index) and the pupae per person index can be calculated from these data.^31^ Additionally, a classification of potential breeding sites, positivity for larvae/pupae, and pupae productivity is calculated. This information can be presented graphically by time period, neighborhood, and sector.

#### Social

A KAP survey was developed and carried out within the context of the project. The instrument consisted of 76 questions, divided into the following blocks: (1) identification and sociodemographic characteristics; (2) characteristics of the home, access to services, and water use; (3) knowledge, attitudes, and practices about dengue; and (4) forms of community organization and access to information. The survey was carried out by the ETV technicians from each of the municipalities. Approximately 400 interviews were carried out per municipality trying to cover most of the neighborhoods of each city. A descriptive analysis, in the form of the proportions of participants who responded for each category for each question, was carried out and compared between the participating municipalities. The survey was approved by the Ethical Review Board of the International Center for Medical Research and Training (*Centro Internacional de Entrenamiento e Investigaciones Medicas*, or CIDEIM).

#### Risk Stratification

Dengue indicators are constructed based on variables that are first standardized to mean zero and standard deviation 1 (*z* scores), then summed to give the following 4 components: (1) epidemiology, which includes cumulative incidence, percentage of cases that were severe, and number of months with 5 or more cases; (2) entomology, which includes the Breteau and pupae/person indices; (3) environment, which includes the number of containers per thousand population; and (4) demography, which includes population density and the number of sites of high concentration of people (e.g., schools, malls, churches). The sites are chosen to reflect mostly daytime activity, which is when the *Aedes* vectors prefer to feed.

For each component, all the values of the standardized variables by neighborhood were summed (with equal weights) and then this sum was divided by the number of variables of each component. Finally, the 4 values, 1 for each component, are summed to obtain a final value for each neighborhood. The results are visualized as quintiles for a given time period, municipality, and event.

#### Action Plans and Follow-Up

This module supports planning activities and suggests priority neighborhoods for 2 scenarios—endemic and outbreak. The scenarios are defined according to whether the number of cases reported during the previous 3 weeks in the municipality was over the 50th percentile of the endemic channel. The software alerts secretariat officials when neighborhoods have a higher rate of disease transmission, in order to facilitate action plans, without excluding actions for other neighborhoods. The software allows for planning activities by neighborhood and following their development. No automatic alerts have yet been created for the system.

The software alerts secretariat officials when neighborhoods have a higher rate of disease transmission in order to facilitate action plans.

### Ethical Statement

The study protocol and surveys was approved by the CIDEIM Ethical Review Board for studies involving human subjects and conducted in accordance with national and international regulations and standards for research involving human participants.

## OBSERVATIONS, EXPERIENCES, AND LESSONS LEARNED

**FIGURE 4:**
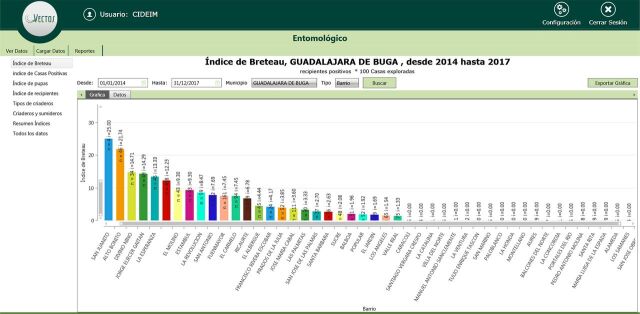
VECTOS Graphical Output of Entomological Breteau Index^a^ for Each Neighborhood in Buga, Colombia, January 1, 2014–December 31, 2017 ^a^Number of positive containers per 100 houses.

VECTOS identified differences by neighborhood in the main mosquito breeding sites and their pupal productivity (data not shown). In particular, findings showed that many neighborhoods in the 3 municipalities are at low risk of mosquito-borne disease transmission, in terms of the Breteau index (number of positive containers per 100 houses) of less than 4 positive containers per 100 houses, or even free of larval breeding sites (Breteau index=0), suggesting responsiveness of their populations to public health messages (Figure 4).

**FIGURE 5:**
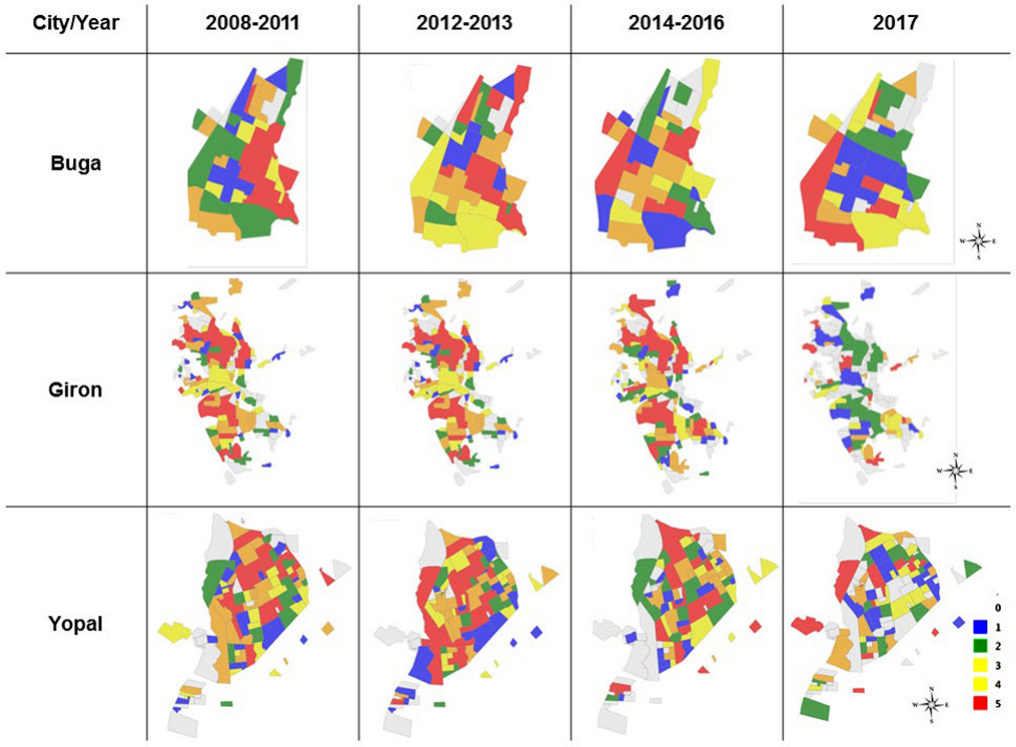
Trends in the Quintiles of the Dengue Epidemiological Risk Component in Buga, Giron, and Yopal, Colombia, 2008–2017 Maps of quintiles of risk stratification, with 1 (blue) being the lowest and 5 (red) being the highest. 0 indicates missing data.

**FIGURE 6:**
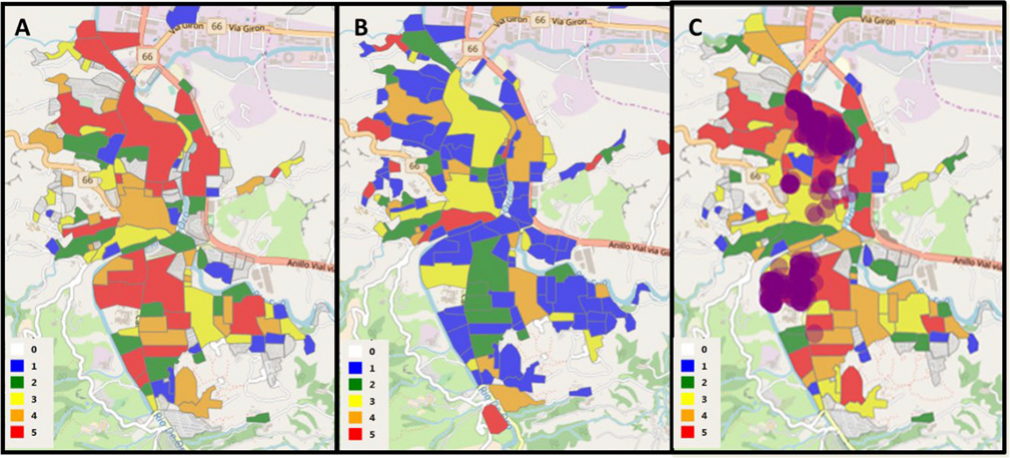
Epidemiological (A) and Entomological (B) Dengue Risk Maps and (C) Location of Positive Catch Basins^a^ in Giron Neighborhoods, Colombia, January 1, 2014–December 31, 2016 ^a^Indicated by purple dots over the epidemiological risk map. Risk stratification in the maps is classified in quintiles, with 1 (blue) being the lowest risk and 5 (red) being the highest, during the observed period. 0 (white) indicates missing data.

During the risk stratification analysis, when we did the epidemiological analyses in the 3 municipalities, for 2008–2016, we observed that some neighborhoods were consistently at high risk during this period, particularly in Giron (Figure 5). When we compare the epidemiological and entomological stratification from Giron, some neighborhoods with consistently higher risk showed a lack of obvious association between the epidemiological component and the entomological component (in premises) (Figure 6A and Figure 6B). These results from Giron prompted a search for other larval breeding sites outside the domestic area, which detected, for the first time in this municipality, high levels of *Aedes spp* larvae in catch basins (storm drains) hidden under sidewalks. These catch basins were part of the town's old drainage system, and were only present in the neighborhoods with persistently high epidemiological risk (Figure 6C). On the basis of these results, Giron's vector control personnel applied larvicides twice per month, from May 2016 to date. Our stratification analyses showed that larval control of these catch basins notably changed the epidemiological risk patterns of this municipality (Figure 5, year 2017).

VECTOS identified, for the first time, high levels of larval breeding sites in catch basins below sidewalks in Giron.

**FIGURE 7:**
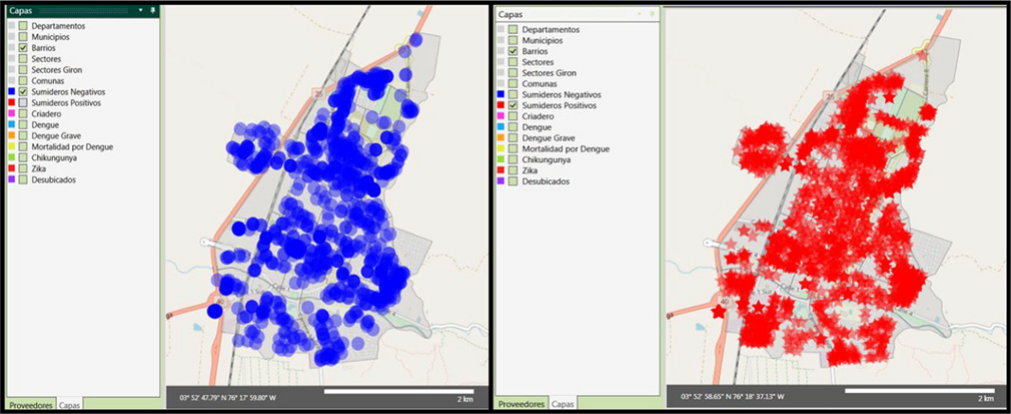
Location of Catch Basins Surveyed in Buga, Colombia, September 2017 Negative catch basins (*Sumideros Negativos*) indicated by blue dots and positive catch basins (*Sumideros Positivos*) indicated by red stars.

A different situation was observed in Buga, where high variation by year in neighborhood-level risk was observed (Figure 5). In Buga, few neighborhoods had entomological risk (Breteau index of more than 4) (Figure 4). However, as we previously reported, almost the entire city can be affected by the presence of positive catch basins (Figure 7, red stars), although control measures are available.^13^

In the social component, surveys were conducted in Buga (372 homes), Giron (437 homes), and Yopal (431 homes) municipalities. The detailed findings will be reported separately. Residents of the 3 municipalities tended to agree that dengue is transmitted by mosquitoes (ranging from 81% to 87%), which bite throughout the day (41% to 54%), and whose reproduction is associated with water (85% to 93%). In terms of disease, the most frequently mentioned symptoms were fever, malaise, and joint pain, usually in that order. However, a proportion of people associated dengue with the common cold and with direct interpersonal transmission (1% to 6%). Interestingly, 12% of residents of Buga and Giron and 4% of residents in Yopal associated the presence of mosquitoes with poor hygiene. Buga was the municipality with the lowest Breteau index per neighborhood (Figure 4). Across the municipalities, few people identified the elimination of larval habitats as a control option (14% to 15%). Insecticide application was the most frequently identified option (58% to 70%), followed by the use of fans to avoid mosquito bites (23% to 43%). The numbers per neighborhood were not sufficient for robust analysis at this level.

These results confirm that risk stratification at the neighborhood level varies between and within cities. Analysis at this scale enabled us to identify neighborhoods with higher epidemiological risk that were persistent in time, as in Giron, and compare this risk with the entomological risk using standard larval indices in houses. This confirmed that cryptic larval habitats were influencing transmission. This is consistent with the paradigm proposed by the DENTARGET network, that such heterogeneity should be recognized in the design of focal dengue control campaigns.^10^ The observed risk stratification demonstrated the importance of a better joint understanding of entomological risk factors and epidemiological patterns.

The integration of information led stakeholders to recognize the importance of improving primary data for decision making. For the epidemiological component, some health care institutions realized the importance of the primary case data for decision making, and an improvement was observed in the completeness and quality of these data, in terms of more complete case reporting, for example, in recording of residential addresses. For the entomological component, the Mosquitos app was developed in consultation with the technicians, which enabled it to facilitate field data collection and analysis, rather than being a technological hurdle. Moreover, analysis of the entomological data confirmed the technicians' personal assessments and enhanced their managers' appreciation of their work. More active participation among stakeholders resulted in a larger sample size and more comprehensive and accurate information. In particular, it enabled us to increase the sample size—from approximately 100 to 1,500 houses per survey per municipality—and hence achieve more comprehensive and accurate information. Unfortunately, other important entomological surveys, such as adult index (mosquitoes per house or area), are not among the activities carried out by the ETV technicians, which would improve entomological analysis and identification of cryptic breeding sites.

The social app (VECTOS Social) was implemented by the technicians who were already familiar with Mosquitos app. The major limitation was the length of each interview (approximately 20 minutes), but we are working to prioritize the most relevant questions for future surveys. We expect that the design of vector control strategies will also benefit from analyses of the social component since this will facilitate the design of more educationally oriented campaigns with better communication strategies. There were few technical limitations in terms of telecommunications, due to generally good wireless data service in the cities. Only a few peripheral locations had sporadic connections, but the apps were designed to cope with this.

However, there are still limitations to be addressed in terms of routine application of the system. These limitations include infrequent updating of municipal planning information in terms of neighborhood definitions and numbers of inhabitants; an unclear legal process required for the national heath entities (Colombian Ministry of Health and Social Protection and National Institute of Health) to authorize technological tools to capture official information; lack of knowledge and use of the software components among officials; and inadequate primary data collection in terms of frequency, sample size, and design of vector control strategies based on the analyses.

The development of VECTOS and the inclusion of epidemiological data from 2008 to 2017 and entomological and social information collected via apps revealed risk patterns not previously identified by the municipalities. In particular, we were able to identify neighborhoods at persistently high epidemiological risk, especially for the municipality of Giron (Figure 6). Such patterns were not readily discernible from previous routine activities.

## CONCLUSION

Despite the limitations of the routinely collected data, their integration into the VECTOS system, including improvements to the data collection procedures, resulted in improved decision-making. Further improvements to data collection and analysis, and presenting the findings in a timely and interpretable fashion, will yield additional benefits to decision makers. Identification of risks in the 3 municipalities enabled us to identify neighborhoods with higher transmission risk that require focal intervention. The current version of VECTOS yields basic indicators over time and space, but future versions could collect additional variables and be linked to mathematical modeling to derive, for example, early warnings of increased transmission, alerts of increased risk of severe illness due to changing serotype profile, and mosquito abundance thresholds for transmission. VECTOS was developed in participation with municipalities from different regions of Colombia with different cultural features, and we expect that this breadth of experience will further strengthen the vector control programs' ability to make evidence-based decisions, and that the lessons learned will be more easily shared across regions.

## Notes

**En español**

**VECTOS: Un Sistema Integrado para el Monitoreo de los Factores De Riesgo Asociados a la Transmisión de Arbovirosis Urbanas**

Con el propósito de fortalecer la vigilancia local de las enfermedades virales transmitidas por mosquitos como el dengue y Zika, un equipo multidisciplinario desarrolló un sistema web de información llamado VECTOS que captura datos entomológicos, epidemiológicos y sociales georeferenciados. El sistema ha revelado características no identificadas previamente, como vecindarios específicos con un riesgo persistentemente alto.

**RESUMEN**

En Colombia, como en muchos países latinoamericanos, la toma de decisiones y el desarrollo de estrategias efectivas para el control de vectores de enfermedades urbanas como el dengue, el Zika y el chikungunya son un desafío para las autoridades locales de salud. La heterogeneidad de la transmisión en las zonas urbanas requiere una asignación eficiente de recursos para implementar las medidas de control basadas en el riesgo. Con el objetivo de fortalecer la capacidad de los sistemas locales de vigilancia para identificar variables que favorecen la transmisión arboviral urbana, un equipo de investigación multidisciplinario colaboró con los funcionarios locales de la Secretaría de Salud de 3 municipios de Colombia (Girón, Yopal y Buga) en el diseño de un sistema de información integrado llamado VECTOS del 2015 al 2018. Tecnologías de información y comunicación se utilizaron para desarrollar 2 aplicaciones móviles para capturar información entomológica y social, así como un sistema basado en la web para la recopilación, la referenciación geográfica y el análisis integrado de la información utilizando softwares geoespacial gratuitos. Este sistema facilita la captura y el análisis de la información epidemiológica del sistema de vigilancia nacional colombiano (SIVIGILA), las encuestas entomológicas periódicas (larvas y pupas de mosquitos en los predios y los sitios de reproducción peridomésticos), y las encuestas de conocimientos, actitudes y prácticas (CAP) en un espacio y contexto temporal a nivel de vecindario. Los datos recopilados en VECTOS se mapean y visualizan en informes gráficos. El sistema permite el monitoreo en tiempo real de indicadores epidemiológicos semanales, índices entomológicos y encuestas sociales. Además, el sistema permite la estratificación de riesgo de los vecindarios, utilizando variables epidemiológicas, entomológicas, demográficas y ambientales seleccionadas. Este artículo describe el sistema VECTOS y las lecciones aprendidas durante su desarrollo y uso. El análisis conjunto de los datos epidemiológicos y entomológicos dentro de un sistema de información geográfica en VECTOS ofrece una mejor comprensión de los datos recopilados de forma rutinaria e identifica la heterogeneidad de los factores de riesgo entre vecindarios. Esperamos que el sistema continúe fortaleciendo los programas de control de vectores en la toma de decisiones basadas en la evidencia y en el diseño y el seguimiento mejorado de las estrategias de control de vectores.

**Peer Reviewed**

**Funding:** This project was financed through the program “Desarrollo investigación aplicada para contribuir a un modelo efectivo y sostenible de intervención del dengue en Santander, Casanare y Valle del Cauca” (Development of applied research to contribute to an effective and sustainable model of dengue intervention in Santander, Casanare and Valle del Cauca), financed by “Sistema General de Regalías (SGR), Fondo de Ciencia, Tecnología e Innovación (CTeI) aprobado por el Órgano Colegiado de Administración y Decisión (OCAD), mediante el Acuerdo 009 de 2013.” Additional support for the Doctoral training of Neila Julieth Mina was received by the Colciencias Fellowship 567-2013 and US NIH International Fogarty Center Global Infectious Disease Research Training Program, Award Number D43 TW006589.
